# Solving Maxwell’s Equations Using Polarimetry Alone

**DOI:** 10.1021/acs.nanolett.4c01976

**Published:** 2024-07-01

**Authors:** Jorge Olmos-Trigo

**Affiliations:** Departamento de Física, Universidad de La Laguna, Apdo. 456, E-38200 San Cristóbal de La Laguna, Santa Cruz de Tenerife, Spain

**Keywords:** Electromagnetism, Nanophotonics, Scattering Theory, Polarization of Light

## Abstract

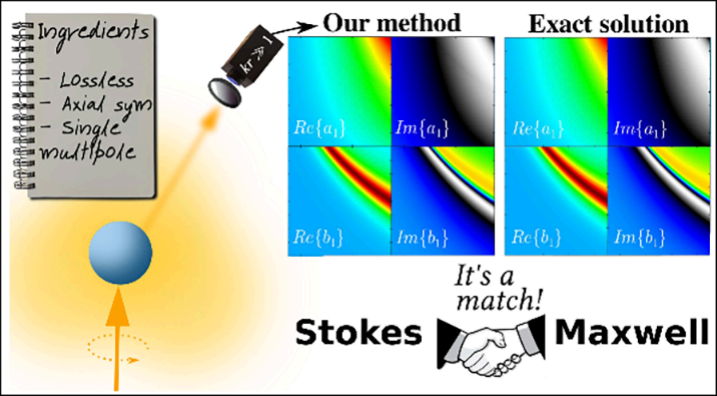

Maxwell’s equations are solved when the amplitude and phase of the electromagnetic field are determined at all points in space. Generally, the Stokes parameters can only capture the amplitude and polarization state of the electromagnetic field in the radiation (far) zone. Therefore, the measurement of the Stokes parameters is, in general, insufficient to solve Maxwell’s equations. In this Letter, we solve Maxwell’s equations for a set of objects widely used in Nanophotonics using the Stokes parameters alone. These objects are lossless, axially symmetric, and well described by a single multipolar order. Our method for solving Maxwell’s equations endows the Stokes parameters an even more fundamental role in the electromagnetic scattering theory.

The determination of the amplitude and phase of the electromagnetic field at all points in space solves Maxwell’s equations.^1^ Currently, electromagnetic software packages can provide the numerical solution to Maxwell’s equations.^2^ However, solving Maxwell’s equations in the optical laboratory is nearly infeasible. One needs to measure the components of the scattered field at all points of the radiation zone. On top of that, the internal field induced in the excited object is experimentally inaccessible. In stark contrast, the Stokes parameters can be readily measured using a photodiode and waveplates.^3,4^ The Stokes parameters can capture the amplitude and polarization state of the electromagnetic field in the radiation (far) zone. Following the notation of ref (4), we can write the Stokes parameters as
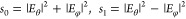
1
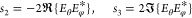
2As eqs 1 and 2 show, the Stokes parameters depend on the transversal components of the scattered electromagnetic field evaluated in the radiation (far) zone, i.e., *E*_θ_ and *E*_φ_. (Note that the longitudinal component of the scattered electromagnetic fields identically vanishes in far-field, namely, *E*_*r*_ = 0.) If the amplitude and phase of *E*_θ_ and *E*_φ_ are measured, the Stokes parameters can be calculated using eqs 1 and 2.^5,6^ The converse is not generally true. To demonstrate this fact, we now write  and , where ξ_θ_ and ξ_φ_ are real-valued phases. Taking into account this notation, we can obtain the following relations from eqs 1 and 2
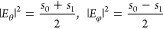
3
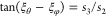
4On the one hand, eq 3 reveals that the measurement of *s*_0_ and *s*_1_ grants access to the amplitudes |*E*_θ_| and |*E*_φ_|. On the other hand, eq 4 shows that measuring *s*_2_ and *s*_3_ provides the phase difference ξ_θ_ – ξ_φ_ but falls short of providing the individual phases ξ_θ_ and ξ_φ_. The determination of the phase of the scattered field is necessary to solve Maxwell’s equations. Therefore, one could conclude that measuring the Stokes parameters is insufficient to solve Maxwell’s equations.

In this Letter, we demonstrate that a measurement of the Stokes parameters at a single scattering angle is sufficient to solve Maxwell’s equations for a set of objects. These objects share the following features: they are lossless, axially symmetric and their optical response is well-described by a single multipole order. Notably, several works have tackled such objects in different branches of Nanophotonics. Examples include optically resonant nanoantennas,^7−11^ Kerker conditions,^12−15^ surface-enhanced Raman scattering,^16^ surface-enhanced optical chirality,^17,18^ among many others.^19−22^ Note that refs (7−22) are experimental studies widely recognized by the Nanophotonics community.

The key to our procedure lies in linking the measurement of the Stokes parameters in the radiation (far) zone with the electric and magnetic scattering coefficients of the multipolar expansion of the scattered field. As we show, the determination of these coefficients solves Maxwell’s equations at all points of the radiation zone, ranging from far-to-near field. Additionally, in the case of spherical objects, we solve Maxwell’s equations at all points in space (also inside the object).

We now consider the scattered **E**_sca_(*k***r**) and internal **E**_int_(*k***r**) electromagnetic fields produced by an arbitrary object. In the usual basis of electric and magnetic multipoles,^23^ we can write the scattered and internal electromagnetic fields as
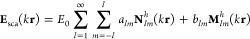
5
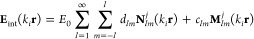
6Here  and ,  and  are spherical Hankel and Bessel functions of the first kind, respectively, (θ, φ) represents the usual vector spherical harmonics,^23^, where *s* = {*j*, *h*}, and  and *m* denote the multipolar order and the total angular momentum, respectively. Moreover **r** = {*r*, θ, φ} is the observational point, *k* is the radiation wavenumber, *k*_*i*_ = m*k*, m being the refractive index of the object, and *E*_0_ is the amplitude of the incident wavefield. Importantly,  and  denote the electric and magnetic scattering coefficients, respectively, and  and  are the internal electric and magnetic coefficients, respectively.

Equations 5 and 6 show that the determination of the set {, , , } grants access to the amplitude and phase of both the scattered and internal electromagnetic fields at all points of space. However, capturing the set {, , , } is exceptionally demanding due to the need to measure the components of the scattered and internal electromagnetic fields in all directions.^23^ In fact and to the best of our knowledge, none of the magnitudes of the set has been experimentally measured.

As previously mentioned, the Stokes vector **S** = {*s*_0_, *s*_1_, *s*_2_, *s*_3_} can be measured in the radiation (far) zone with conventional optical components such as a photodiode and waveplates. Hereafter, we consider objects well-described by fixed values of *m* and . In other words, we deal with axially symmetric objects whose optical response is described by a single multipolar order. We recall that such objects have been widely studied in Nanophotonics.^7−20,20−22^ In this setting ( is fixed), let us insert the far-field limit (*kr* → *∞*) of eq 5 into eqs 1 and 2). After some algebra (see Supporting Information S1), we get^24^
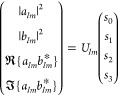
7Equation 7 shows that all the quadratic combinations of {, } can be attained from a single Stokes vector measurement in the far-field. As proved in ref (24), all that one needs to do is compute the 4 × 4 matrix  and apply it to the Stokes measurement. However, we anticipate that even if the object is well-described by fixed values of *m* and , the phases of  and  cannot be attained using eq 7. To prove it, we now write  and , where ϕ_*a*_ and ϕ_*b*_ are real-valued phases. In this setting, the last two rows of the left side of eq 7 can be manipulated to yield
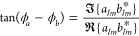
8Equation 8 provides access to the phase difference ϕ_*a*_ – ϕ_*b*_ but not to the individual phases ϕ_*a*_ and ϕ_*b*_. Consequently, without knowledge of these individual phases, determining the scattering coefficients  and  becomes impossible. Due to this infeasibility, we cannot capture the scattered field **E**_sca_(*k***r**) using eq 7, and the solution to Maxwell’s equations is not attained.

In the forthcoming, we show that the phase-indetermination of eq 8 is resolved if the light-scattering system is lossless.

We now consider the extinction and scattering cross sections, denoted by σ_ext_ = σ_ext_^e^ + σ_ext_^m^ and σ_sca_ = σ_sca_^e^ + σ_sca_^m^, respectively. Note that here e and m denote the electric and magnetic contributions, respectively. The extinction and scattering cross sections can be written as^25^
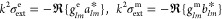
9
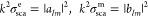
10Here  and  denote the beam-shape coefficients of the incident wavefield.^26^ Objects without optical losses satisfy σ_ext_ = σ_sca_. According to eqs 9 and 10, lossless objects fulfill
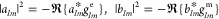
11Equation 11 shows that the amplitude of the electric (and magnetic) scattering coefficient is a function of the electric (and magnetic) phase.^27−29^ That noted, by expanding eq 11 and manipulating eq 8, we arrive to
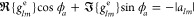
12
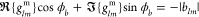
13
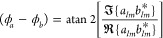
14We now reach notable results. The system of eqs 12–14 can be unambiguously solved yielding ϕ_*a*_ and ϕ_*b*_ (in the correct quadrant). Note that the right-side of eqs 12–14 can be obtained using eq 7. Moreover, the beam-shape coefficients  are usually known quantities since the incident electromagnetic field can be controlled. Now, capturing ϕ_*a*_ and ϕ_*b*_ along with the amplitudes || and || allows us to determine the electric and magnetic scattering coefficients  and . As eq 5 shows, the determination of  and  grants access to all the components of the scattered field evaluated at all points of the radiation zone, ranging from far-to-near-field. In simple words, the determination of  and  solves Maxwell’s equations in the radiation zone.

At this point, let us highlight the main features of our method for solving Maxwell’s equations in the radiation zone:

- Simplicity in the measurement: Our method relies on a measurement of the Stokes parameters at a single angle. From an experimental standpoint, we must avoid any propagation direction where the incident wavefield has a component. Otherwise, the total field measured in the optical laboratory will be the sum of the scattered and incident wavefields, thus invalidating our method.
- Generality of the material and shape of the object: Our method works for axially symmetrical objects such as disks, pillars, spheres, and spheroids. Note that these objects can be composed of a single material or not (coated objects). The only requirement is that the absorption cross-section of such objects must be zero.
- Wide range of illumination conditions: Our method can accommodate plane waves, Gaussian beams,^36^ or even vortex wavefields.^26,30^ The only requirement is that the total angular momentum *m* of the light-scattering system must be well-defined.

At this point, let us illustrate the relevance of eqs 12–14 with one of the most canonical examples used in Nanophotonics: all-dielectric spherical nanoparticles excited by a plane wave. For details on the beam-shape coefficients of a plane wave, check Supporting Information S2. Additionally, check Supporting Information S3 to learn how eqs 12–14 simplify for spherical and lossless particles.

First, we illustrate the accuracy of our method to capture *a*_1_ and *b*_1_. In Figure 1, we depict , , , and  calculated from a Stokes measurement at the scattering angle θ = 90° and utilizing Mie theory (exact solution). Note that φ does not play a role due to the symmetries of the light-scattering system. Additionally, notice that other scattering angles θ could have been selected. The calculation of the Mie coefficients obtained from the Stokes measurement shows an excellent agreement with the exact solution in the broadband interval of refractive index contrasts 2 < m < 4 and optical sizes 0.6 < *x* < 1. Here x = *ka* = 2πa/λ, λ and a being the radiation wavelength and the radius of the object, respectively. To get a deeper insight, the percentage relative error between the exact solution and our novel procedure, as summarized in eqs 12–14, is shown Figure 1 i-l). As previously stated, the overall agreement is remarkable. Note that the significant error in Figure 1) arises since our novel procedure captures  at slightly different spectral points compared to the exact solution.

**Figure 1:**
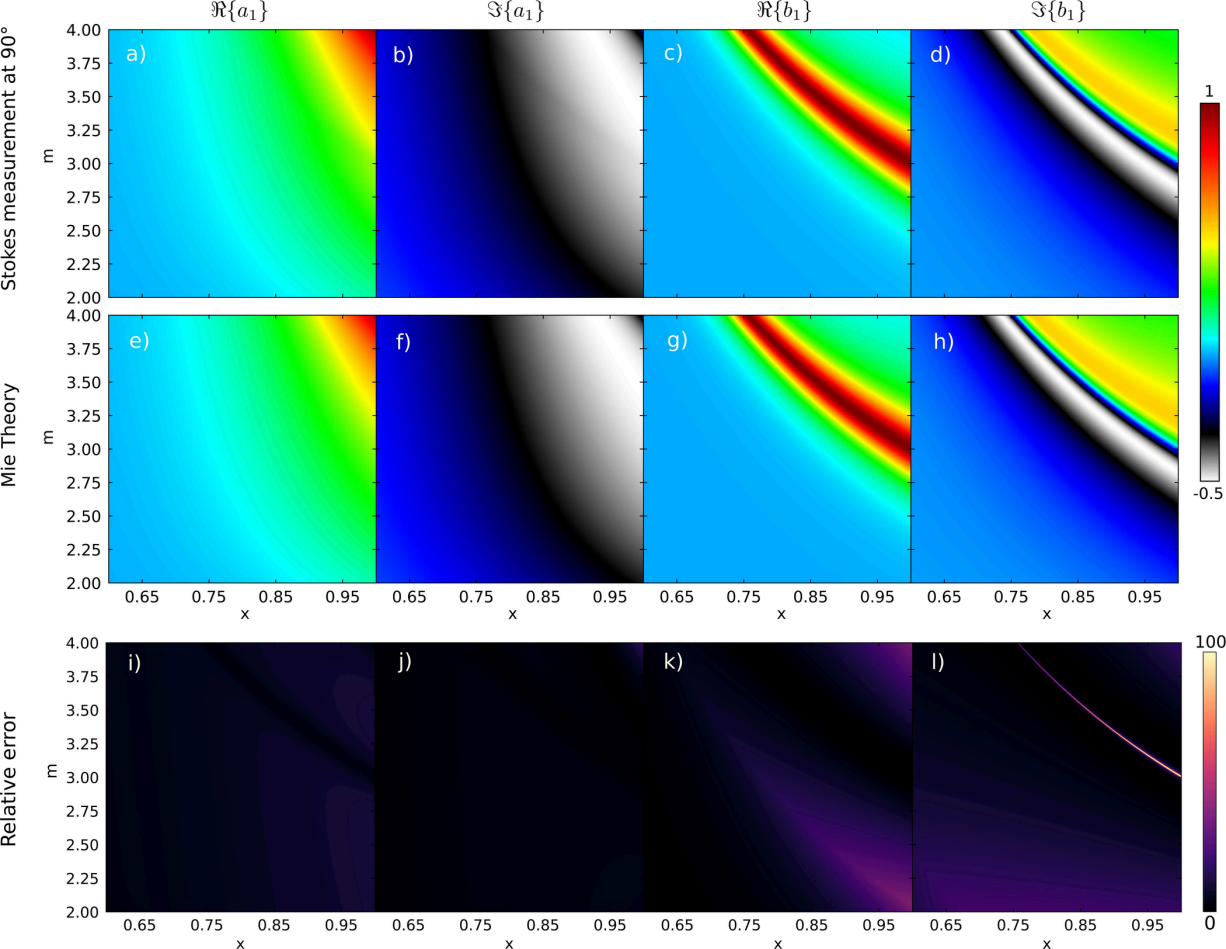
Real and imaginary parts of the dipolar electric and magnetic Mie coefficients obtained from both a Stokes measurement at θ = 90° (see a–d) and using exact Mie theory (see e–h). The excitation wavefield is a circularly polarized plane wave in both cases. The real and imaginary parts of the Mie coefficients are depicted vs the refractive index contrast m and the optical size *x* = *ka* = (2π*a*)/λ, λ and *a* being the radiation wavelength and the radius of the spherical nanoparticle. The intense red colors indicate the Mie resonances. The percentage relative error (i–l) calculated from using Mie theory and the Stokes measurement at θ = 90° is also shown to gain further insights.

Note that for 0 < *x* < 0.6, our approach works as it holds for objects described by an electric and/or magnetic response. We stress that the scattering Mie coefficients  and  do not depend on the incident illumination. Therefore, once we determine  and  using eqs 12–14 for a specific illumination, such as a plane wave, we can subsequently explore the scattering features of the spherical object under general illumination conditions.

Interestingly, the dipolar Mie coefficients are biunequivocally determined by the electric and magnetic polarizabilities, often denoted as α_E_ and α_M_, respectively. For the sake of clarity, let us write the correspondence between Mie coefficients and polarizabilities^31^
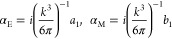
15Equation 15 can be calculated from eqs 12–14, and thus, our Stokes method can be employed to retrieve the electric and magnetic polarizabilities when dealing with dipolar Mie objects.

At this point, we show the accuracy of our method to solve the Maxwell equations in the radiation zone with a realistic material. Particularly, we consider a Gallium Phosphide (GaP) nanoparticle of radius *a* = 75 nm excited by a circularly polarized plane wave.^11^ We select GaP as it is a material with high potential for metasurface-based devices operating across the visible, as it presents a high-refractive index (*m* > 3.3) and negligible losses.^32^

Figure 2a-d shows , , , and  calculated from a Stokes measurement at θ = 90° and θ = 60° and employing Mie theory. The scattering Mie coefficients calculated from the Stokes measurements at the specified angles exhibit excellent agreement with the exact calculations (and with each other) in the range 475 nm < λ < 700 nm.

**Figure 2:**
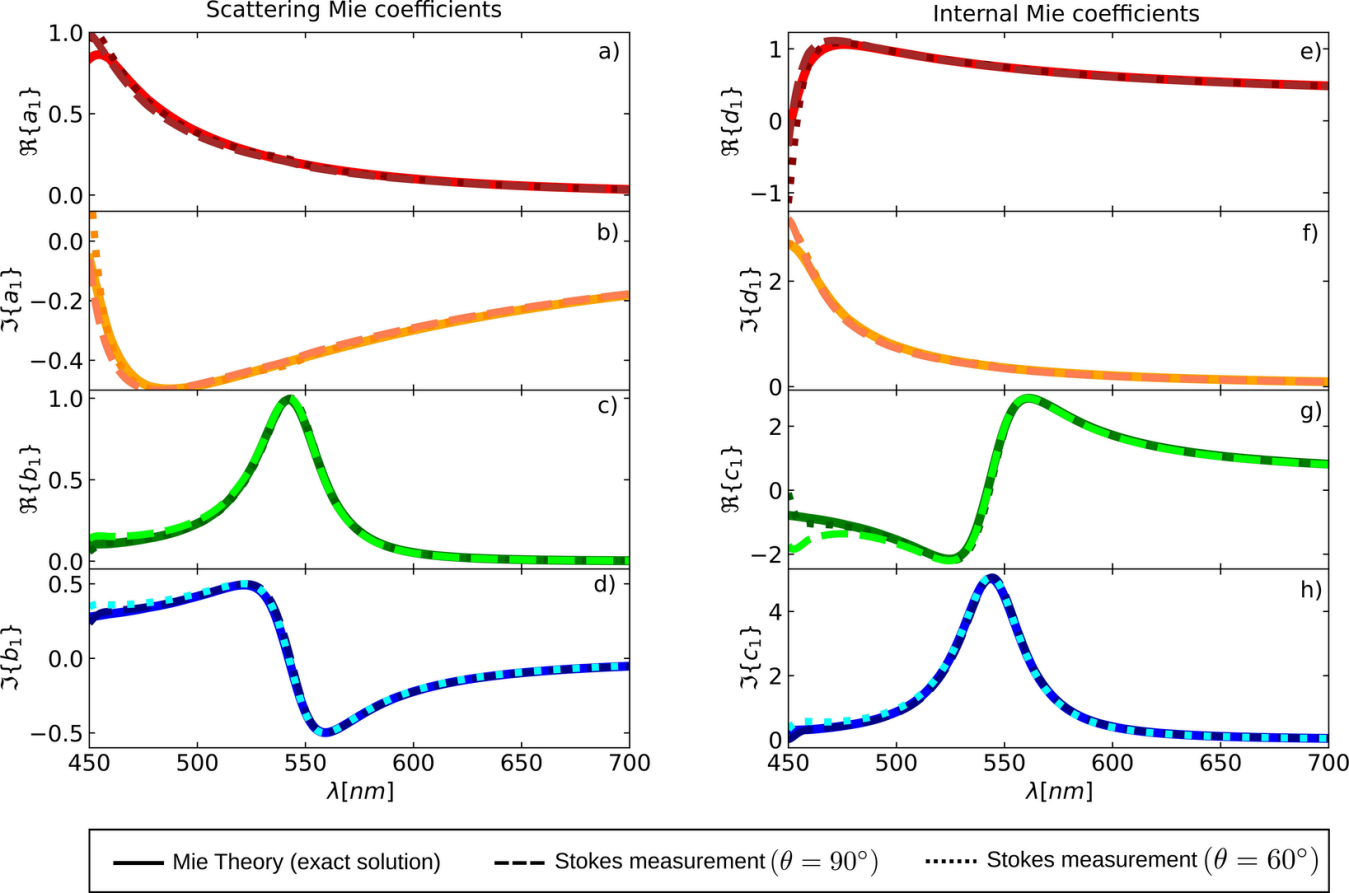
Real and imaginary parts of the scattering (see a–d) and internal (see e–h) Mie coefficients of a GaP spherical object with radii *a* = 75 nm obtained from both a Stokes measurement at θ = 90° (dashed) and θ = 60° (dotted). The excitation wavefield is a circularly polarized plane wave in all cases. The scattering and internal Mie coefficients are depicted vs the incident wavelength λ.

It is worth noting that the results obtained from the Stokes vector measurement exhibit slight deviations from each other (and from the exact result) at shorter wavelengths, specifically in the range 450 nm < λ < 475 nm. This deviation arises because, in this wavelength range, the scattering cannot be fully described by  = *m* = 1 due to the presence of the magnetic quadrupole. In addition to this, GaP presents non-negligible optical losses for λ < 475 nm.^33^ Due to these facts, our method, as summarized in eqs 12–14, cannot be applied for shorter wavelengths λ < 475 nm since the setting we assumed—a lossless dipolar object—is not fulfilled.

Importantly, our Stokes method robustly detects that the setting we assumed does not fully hold as the curves for θ = 90° and θ = 60° deviate in the range 450 nm < λ < 475 nm.

We now summarized the scope of the method. Our Stokes method is reliable if the calculated coefficients remain identical regardless of the scattering angle θ of the collection. In this scenario, one can experimentally retrieve the full solution of Maxwell equations. If such coefficients differ, then the scattering cannot be fully described by the selected values of  and *m* in the  matrix or/and the light-scattering system is not lossless. In other words, the Stokes method is robust and can be trusted upon measuring the Stokes parameters at different scattering angles of collection.

In Supporting Information S5, we discuss the role of optical losses and higher multipolar orders in the accuracy of the Stokes method. In particular, we consider a different GaP nanosphere at shorter wavelengths, where losses are high.

Next, we show that the determination of the scattering Mie coefficients grants access to the internal Mie coefficients.

In 1908, Gustav Mie solved the scattering of a plane wave by a spherical object.^34^ Specifically, Mie determined the scattering {, } and internal coefficients {, }. The relation between {, } {, } and can be compactly written as^4^
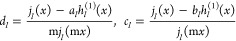
16Equation 16 shows that the internal Mie coefficients {, } can be determined from the scattering coefficients {, }. Thus, the internal Mie coefficients can be captured using eqs 12–14 particularized for spherical particles.

In Figure 2, we plot , , , and  using eq 16. For this calculation, we have employed *a*_1_ and *b*_1_, which, in turn, have been previously obtained using eqs 12–14 at θ = 90° and θ = 60°. As could be expected, the calculation of the internal Mie coefficients shows an excellent agreement with the exact calculation in the wavelength interval 475 nm < λ < 700 nm.

As mentioned in the introduction, the determination of internal and scattering coefficients gives rise to the exact solution to Maxwell’s equations. Since every electromagnetic physical magnitude originates from the electromagnetic field, we can also access, for instance, the exact internal dipolar moments, denoted as **p** and **m** in ref (35).

In conclusion, we have presented a method that solves Maxwell’s equations at all points in the radiation zone based on a single measurement of the Stokes parameters in the far-field. We have illustrated the accuracy of our method with one of the most studied systems in Nanophotonics: a spherical nanoparticle excited by a plane wave. In this setting, we have also determined the internal Mie coefficients, solving Maxwell’s equations at all points in the space.

To the best of our knowledge, this study represents the first method capable of solving Maxwell’s equations experimentally and from a measurement of the Stokes parameters. This feature endows the Stokes parameters, mostly used to get insight into the polarization state of the electromagnetic radiation, an even more fundamental role in the electromagnetic scattering theory. Consequently, our findings, supported by analytical theory and exact numerical simulations, can find applications in all branches of Nanophotonics and Optics.

## Supporting Information Available

S1: The Stokes method used to derive eq 7. S2: The beam shape coefficients of a plane wave. S3: The Stokes method applied to a magnetodielectric spherical nanoparticle. S4: The role of optical losses and higher multipolar orders in the accuracy of solving Maxwell’s equations from the Stokes method (PDF)
