# Correction to “Solving Maxwell’s Equations Using Polarimetry Alone”

**DOI:** 10.1021/acs.nanolett.4c03869

**Published:** 2024-09-03

**Authors:** Jorge Olmos-Trigo

**Funding Information.** The funding information should read as:

*“J.O.-T. acknowledges support from the Juan de la Cierva-Formación fellowship No. FJC2021–047090-I and acknowledges financial support from the Spanish Ministry of Science and Innovation (MCIN), AEI, and FEDER (UE) through projects PID2022–137569NB-C43 and PID2022–143268NB-I00.”*

**References to Historical Contributions.** The historical contributions to the local and averaged Stokes parameters should be more explicitly stated.

In 2019, we demonstrated that a local measurement of *s*_0_ and *s*_3_ at 90° provides access to the expected (integrated) value of electromagnetic helicity in the dipolar regime (see eq 8 in ref (1)). Later, in 2023, we discovered that the relationship between *s*_0_ and *s*_3_ and their integrated counterparts, ⟨*s*_0_⟩ and ⟨*s*_3_⟩, holds true for any scattering angle.^2^ Furthermore, we found that this relationship extends to larger spherical objects when excited with structured light, expanding its applicability beyond the dipolar regime.

However, it is important to acknowledge that the results of refs (1 and 2) cannot be used to solve Maxwell’s equations. To start with, the Stokes vector is not measured, making it impossible to disentangle the electric and magnetic scattering coefficients. In addition to this, the imposition of the lossless condition, which is crucial for solving Maxwell’s equations by linking the amplitude and phase of the scattering coefficients (see eq 11 of this work), is also absent in refs (1 and 2).

These new references are listed in the References section below.
