# Supplementary material for: Solving Maxwell’s Equations Using Polarimetry Alone
Source: Nano Lett. 2024 Jul 1;24(28):8658–63. doi: 10.1021/acs.nanolett.4c01976 (PMC11261596; doi:10.1021/acs.nanolett.4c01976)
Supplement: Supplementary file 1 — nl4c01976_si_001.pdf [file nl4c01976_si_001.pdf]

SUPPORTING INFORMATION

Solving Maxwell's Equations Using Polarimetry Alone

Jorge Olmos-Trigo*

Departamento de Física, Universidad de La Laguna, Apdo. 456. E-38200, San Cristóbal de La Laguna, Santa Cruz de Tenerife, Spain.

E-mail: jolmostrigo@gmail.com

S1: The Stokes method

The scattered field $\mathbf{E}(k\mathbf{r})$ in the radiation zone (when $kr \to \infty$) can be expressed as[1]

$$\lim_{kr\to\infty} \mathbf{E}(k\mathbf{r}) = \left[E_\theta \hat{\mathbf{e}}_\theta + E_\varphi \hat{\mathbf{e}}_\varphi\right], \quad (1)$$

where

$$E_\theta = E_0 \sum_{\ell m} \bar{C}_{\ell m}(kr,\varphi)\left[a_{\ell m}\tau_{\ell m}(\theta) - imb_{\ell m}\pi_{\ell m}(\theta)\right], \quad (2)$$

$$E_\varphi = E_0 \sum_{\ell m} \bar{C}_{\ell m}(kr,\varphi)\left[ima_{\ell m}\pi_{\ell m}(\theta) + b_{\ell m}\tau_{\ell m}(\theta)\right]. \quad (3)$$

Here $a_{\ell m}$ and $b_{\ell m}$ denote the electric and magnetic scattering coefficients, respectively, ℓ and m being the multipolar order and total angular momentum, respectively. Additionally, E_0 is the amplitude of the incident light field, k is the radiation wavenumber, $r = |\mathbf{r}|$ denotes the observation distance to the center of the object, and θ and φ denote the scattering and azimuth observational angles, respectively. Moreover, we have used [1]

$$\pi_{\ell m}(\theta) = \frac{P_\ell^m(\cos\theta)}{\sin\theta}, \qquad \tau_{\ell m}(\theta) = \frac{dP_\ell^m(\cos\theta)}{d\theta}, \quad (4)$$

where $P_\ell^m(\cos\theta)$ are the Associated Legendre Polynomials[1] and

$$\bar{C}_{\ell m}(kr,\varphi) = \frac{e^{ikr}}{kr}\left[\frac{(-i)^{\ell+2}}{\sqrt{\ell(\ell+1)}}\sqrt{\frac{2\ell+1}{4\pi}\frac{(\ell-m)!}{(\ell+m)!}}\right]e^{im\varphi}. \quad (5)$$

At this stage, we have introduced all the requirements to calculate the Stokes vector $\mathbf{S} = \{s_0, s_1, s_2, s_3\}$. Now, by considering that the optical response of the object can be described by

[1] $\pi_{lm}(\theta)$ and $\tau_{lm}(\theta)$ are real-valued functions that Bohren and Huffman used to address the absorption and scattering by a sphere for $m = 1$ (see Eq. 4.46 of Ref.[2]).

a single multipolar order ℓ and total angular momentum m, it can be shown that[3]

$$\tilde{s}_0 = (|a_{\ell m}|^2 + |b_{\ell m}|^2)\gamma_{\ell m}(\theta) - 4\Im\{a_{\ell m}b^*_{\ell m}\}\eta_{\ell m}(\theta), \tag{6}$$

$$\tilde{s}_1 = (|a_{\ell m}|^2 - |b_{\ell m}|^2)\nu_{\ell m}(\theta), \tag{7}$$

$$\tilde{s}_2 = -2\Re\{a_{\ell m}b^*_{\ell m}\}\nu_{\ell m}(\theta), \tag{8}$$

$$\tilde{s}_3 = 2\left[\Im\{a_{\ell m}b^*_{\ell m}\}\gamma_{\ell m}(\theta) - (|a_{\ell m}|^2 + |b_{\ell m}|^2)\eta_{\ell m}(\theta)\right]. \tag{9}$$

Here, we have defined $\mathbf{S} = |E_0|^2|\bar{C}_{\ell m}(kr,\varphi)|^2\tilde{\mathbf{S}}$ along with

$$\gamma_{\ell m}(\theta) = \left[\tau^2_{\ell m}(\theta) + m^2\pi^2_{\ell m}(\theta)\right], \tag{10}$$

$$\eta_{\ell m}(\theta) = m\tau_{\ell m}(\theta)\pi_{\ell m}(\theta), \tag{11}$$

$$\nu_{\ell m}(\theta) = \left[\tau^2_{\ell m}(\theta) - m^2\pi^2_{\ell m}(\theta)\right]. \tag{12}$$

Now, we can rewrite Eqs. (6)-(9) as[3]

$$\mathbf{D}_{\ell m} = U_{\ell m}\mathbf{S}, \tag{13}$$

$$U_{\ell m} = \frac{1}{A_{\ell m}}\begin{pmatrix} \gamma_{\ell m} & \nu_{\ell m} & 0 & 2\eta_{\ell m} \\ \gamma_{\ell m} & -\nu_{\ell m} & 0 & 2\eta_{\ell m} \\ 0 & 0 & -\nu_{\ell m} & 0 \\ 2\eta_{\ell m} & 0 & 0 & \gamma_{\ell m} \end{pmatrix}, \tag{14}$$

with $A_{\ell m} = 2|E_0|^2|\bar{C}_{\ell m}|^2\nu^2_{\ell m}$, and

$$\mathbf{D}_{\ell m} = \begin{pmatrix} |a_{\ell m}|^2 \\ |b_{\ell m}|^2 \\ \Re\{a_{\ell m}b^*_{\ell m}\} \\ \Im\{a_{\ell m}b^*_{\ell m}\} \end{pmatrix}, \qquad \mathbf{S} = \begin{pmatrix} s_0 \\ s_1 \\ s_2 \\ s_3 \end{pmatrix}. \tag{15}$$

S2: The beam shape coefficients of a plane wave

The beam-shape coefficients of a plane wave in the z-direction, $\mathbf{E}_{\rm inc} = E_0 e^{ikz}(p_x, p_y, 0)$, are well-known and given by[1]

$$g^{\rm e}_{\ell,\pm 1} = G_\ell\left(\mp i p_x - p_y\right), \qquad g^{\rm m}_{\ell,\pm 1} = G_\ell\left(p_x \mp i p_y\right), \tag{16}$$

where $G_\ell = i^\ell\sqrt{\pi(2\ell+1)}$. The polarization is described by the components of the Jones vector p_x and p_y, which satisfies $|p_x|^2 + |p_y|^2 = 1$. For the sake of simplicity, let us fix the incident wavefield to a circularly polarized plane. A circularly polarized plane wave satisfies $m = p$, where $p = \pm 1$ is the helicity (handedness) of the wave. For instance, a left-handed polarized plane wave carries $p = +1$ and thus $m = +1$. The components of the Jones vector for a left-handed polarized plane wave are given by $p_x = -i p_y = 1/\sqrt{2}$. Hence, the beam shape coefficients of a left-handed circularly polarized plane wave are given by

$$g^{\rm e}_{\ell 1} = -i\bar{G}_\ell, \qquad g^{\rm m}_{\ell 1} = \bar{G}_\ell \tag{17}$$

where $\bar{G}_\ell = \sqrt{2}G_\ell$. These are the beam-shape coefficients used in the main manuscript.

S3: A lossless magnetodielectric spherical nanoparticle

The T-matrix of a spherical particle is diagonal and satisfies $a_{\ell m} = -g^{\rm e}_{\ell m} a_\ell$ and $b_{\ell m} = -g^{\rm m}_{\ell m} b_\ell$, a_ℓ and b_ℓ being the electric and magnetic Mie coefficients, respectively.[4] Taking these relations into

account, we can write from the left side of Eq. 12

$$|a_{\ell m}|^2 = |g^{\mathrm{e}}_{\ell m}|^2 |a_\ell|^2, \tag{18}$$

$$|b_{\ell m}|^2 = |g^{\mathrm{m}}_{\ell m}|^2 |b_\ell|^2, \tag{19}$$

$$\Re\{a_{\ell m} b^*_{\ell m}\} = \Re\{a_\ell b^*_\ell\} \Re\{g^{\mathrm{e}}_{\ell m} g^{\mathrm{m}\,*}_{\ell m}\} - \Im\{a_\ell b^*_\ell\} \Im\{g^{\mathrm{e}}_{\ell m} g^{\mathrm{m}\,*}_{\ell m}\}, \tag{20}$$

$$\Im\{a_{\ell m} b^*_{\ell m}\} = \Re\{a_\ell b^*_\ell\} \Im\{g^{\mathrm{e}}_{\ell m} g^{\mathrm{m}\,*}_{\ell m}\} + \Im\{a_\ell b^*_\ell\} \Re\{g^{\mathrm{e}}_{\ell m} g^{\mathrm{m}\,*}_{\ell m}\}. \tag{21}$$

Now, the electric and magnetic Mie coefficients of a lossless spherical particle can be written in the scattering phase-shift notation.[5] That is,

$$a_\ell = i \sin\alpha_\ell e^{-i\alpha_\ell}, \qquad b_\ell = i \sin\beta_\ell e^{-i\beta_\ell}, \tag{22}$$

where α_ℓ and β_ℓ are real in the absence of losses. In this setting, it can be shown that $\Re\{a_\ell\} = |a_\ell|^2 = \sin^2\alpha_\ell$ and $\Re\{b_\ell\} = |b_\ell|^2 = \sin^2\beta_\ell$. Note that $|a_\ell| > 0$ and $|b_\ell| > 0$ implies that $0 < \alpha_\ell < \pi$ and $0 < \beta_\ell < \pi$, respectively. Inserting Eq. (22) into Eqs. (18)-(21) yield

$$|a_{\ell m}|^2 = |g^{\mathrm{e}}_{\ell m}|^2 \sin^2\alpha_\ell, \tag{23}$$

$$|b_{\ell m}|^2 = |g^{\mathrm{m}}_{\ell m}|^2 \sin^2\beta_\ell, \tag{24}$$

$$\begin{aligned}\Re\{a_{\ell m} b^*_{\ell m}\} &= \sin\alpha_\ell \sin\beta_\ell \cos(\alpha_\ell - \beta_\ell) \Re\{g^{\mathrm{e}}_{\ell m} g^{\mathrm{m}\,*}_{\ell m}\} \\ &\quad + \sin\alpha_\ell \sin\beta_\ell \sin(\alpha_\ell - \beta_\ell) \Im\{g^{\mathrm{e}}_{\ell m} g^{\mathrm{m}\,*}_{\ell m}\},\end{aligned} \tag{25}$$

$$\begin{aligned}\Im\{a_{\ell m} b^*_{\ell m}\} &= \sin\alpha_\ell \sin\beta_\ell \cos(\alpha_\ell - \beta_\ell) \Im\{g^{\mathrm{e}}_{\ell m} g^{\mathrm{m}\,*}_{\ell m}\} \\ &\quad - \sin\alpha_\ell \sin\beta_\ell \sin(\alpha_\ell - \beta_\ell) \Re\{g^{\mathrm{e}}_{\ell m} g^{\mathrm{m}\,*}_{\ell m}\}.\end{aligned} \tag{26}$$

These equations are valid for a lossless spherical particle. For the sake of simplicity, let us insert the beam shape coefficients of a left-handed circularly polarized plane wave (see Eq. (17)) into Eqs. (23)-(26). Moreover, let us assume $\ell = 1$ (dipolar response). Taking into account this setting,

we arrive to

$$|a_{11}|^2 = |\bar{G}_1|^2 \sin^2 \alpha_1, \tag{27}$$

$$|b_{11}|^2 = |\bar{G}_1|^2 \sin^2 \beta_1, \tag{28}$$

$$\Re\{a_{11}b_{11}^*\} = -|\bar{G}_1|^2 \sin\alpha_1 \sin\beta_1, \sin(\alpha_1 - \beta_1), \tag{29}$$

$$\Im\{a_{11}b_{11}^*\} = -|\bar{G}_1|^2 \sin\alpha_1 \sin\beta_1 \cos(\alpha_1 - \beta_1). \tag{30}$$

These equations can be further simplified, yielding

$$|a_{11}| = |\bar{G}_1| \sin\alpha_1, \tag{31}$$

$$|b_{11}| = |\bar{G}_1| \sin\beta_1, \tag{32}$$

$$\text{atan2}\left[\frac{\Re\{a_{11}b_{11}^*\}}{\Im\{a_{11}b_{11}^*\}}\right] = \alpha_1 - \beta_1. \tag{33}$$

These equations can be solved from a single measurement of the Stokes parameters. As we explain in the main text, this Stokes measurement yields a_{11}, b_{11}, $\Re\{a_{11}b_{11}^*\}$, $\Im\{a_{11}b_{11}^*\}$. Then, by using Eq. (31)-(33) we can unambiguously obtain α_1 and β_1. Then, by inserting these values into Eq. (22) we can obtain the electric and magnetic Mie coefficients for $\ell = 1$. This is the procedure that we have followed to calculate Figs 1-2.

S4: The role of optical losses and higher multipolar orders

In this section, we discuss the role of optical losses in the accuracy of the Stokes method to solve Maxwell equations. Now, we know that losses inhibit the relations $\Re\{a_\ell\} = |a_\ell|^2$ and $\Re\{b_\ell\} = |b_\ell|^2$. In addition to this, the electric α_ℓ and magnetic β_ℓ scattering angles are not real for lossy systems. This fact inhibits the relation between the amplitude and phase of the scattering coefficients, written, for instance, in Eqs. (23)-(24). Thus, we anticipate that losses will cause failures to retrieve the solution of Maxwell equations via the Stokes method. The key question is: to

what extent can our Stokes method be used for lossy systems?

We discuss the accuracy of the presented Stokes method in Fig. 1 for a different GaP nanosphere ($R = 60$ nm) at shorter wavelengths (350-700 nm). Note that in Fig. 1a), we plot the refractive index of GaP in this wavelength interval. In Fig. 1b) we depict $\Re\{b_1\}$ and in Fig. 1c) $\Im\{b_1\}$. Two cases are considered: the exact solution provided by Mie theory (solid lines) and the proposed method (dashed lines) derived from the Stokes measurement at 90°. Please note that other angles could be selected.

As shown in Fig. 1, losses cause the proposed Stokes method to give some errors with respect to the exact solution. In particular, the presented Stokes method overestimates the strength of the magnetic resonance, located at $\lambda = 464$ nm. That is, the Stokes method gives $\Re\{b_1\} = 1$ while the exact solution $\Re\{b_1\} = 0.85$. We can now understand this fact. When losses are not negligible, Eqs. (9)-(10) are not fully met, leading Eqs. (10)-(13) to some errors. In other words, when losses are present, $\Re\{b_1\} > |b_1|^2$, and thus the relation between the amplitude and phase of the scattering coefficients (in this case the magnetic) is lost.

In addition to this, the presence of the quadrupole at $\lambda = 375$ nm causes errors in retrieving $\Re\{b_1\}$ for shorter wavelengths. In particular, in the wavelength interval $350-400$ nm. The combined effect of the quadrupole and optical losses becomes even more pronounced in Fig. 1c). For $\lambda < 450$ nm, the Stokes method and the exact solution give completely different curves due to the combination of these effects: quadrupole and losses.

From the information provided in Fig 1, we can conclude that losses give rise to errors in the absolute value of the magnitudes (see the ones caused at the magnetic resonance) while the presence of the quadrupole disrupts the general tendency. However, further investigation might be required to establish general conclusions.

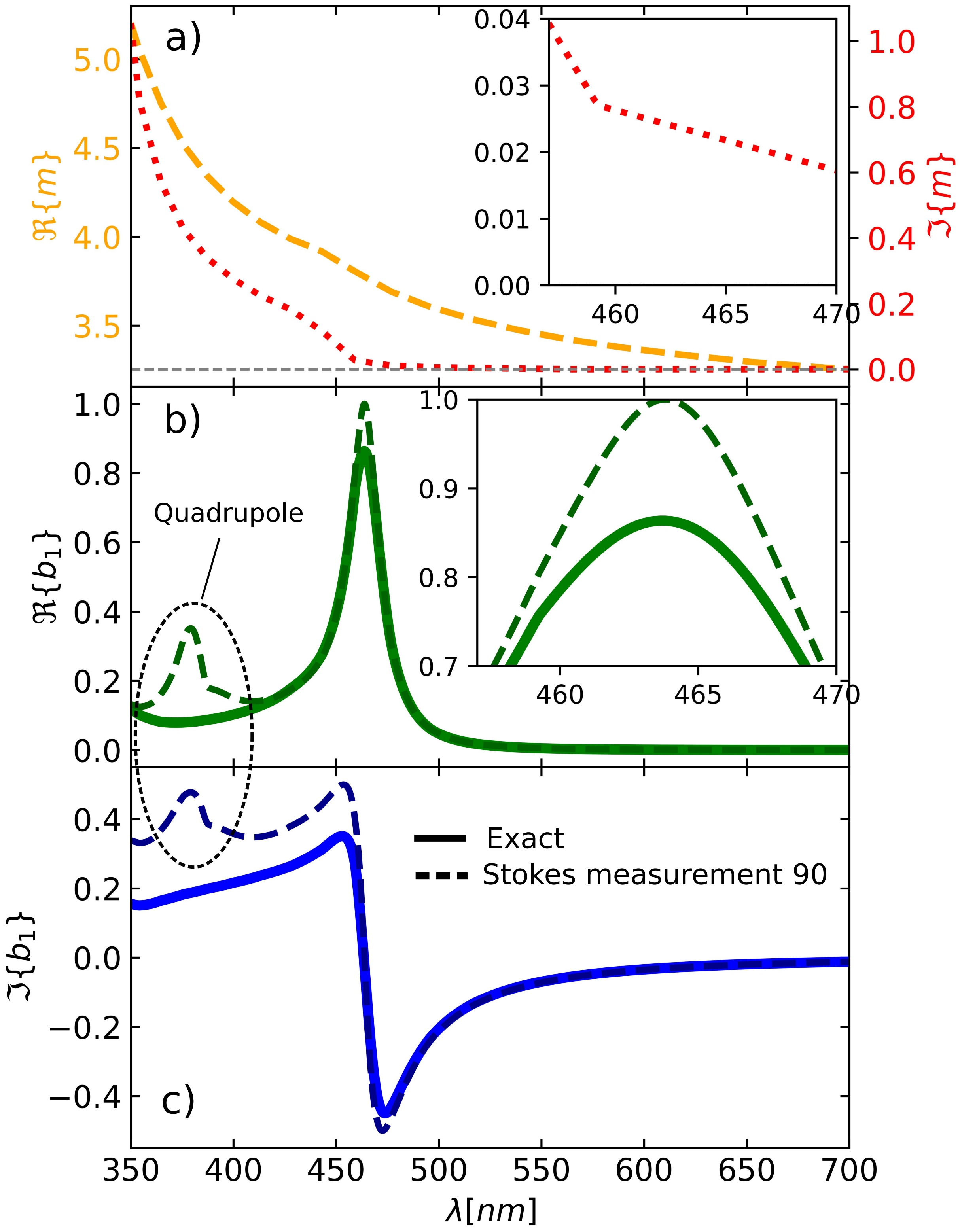

Figure 1: (a) Refractive index of GaP nanosphere ($R = 60$ nm) over the wavelength range of 350-750 nm. (b) Real part of the scattering magnetic coefficient b_1 as a function of wavelength, comparing the exact solution from Mie theory (solid lines) with the proposed Stokes method (dashed lines) at a scattering angle of 90°. (c) Imaginary part of the scattering coefficient b_1 for the same comparison.

References

(1) Jackson, J. D. *Classical Electrodynamics*; John Wiley & Sons, New York, 1999.

(2) Bohren, C. F.; Huffman, D. R. *Absorption and scattering of light by small particles*; John Wiley & Sons, 2008.

(3) Olmos-Trigo, J. The Stokes Vector Measurement: A Paradigm Shift in Electric-Magnetic Light Distinction. *arXiv preprint arXiv:2310.17946* **2023**,

(4) Mie, G. Beiträge zur Optik trüber Medien, speziell kolloidaler Metallösungen. *Annalen der physik* **1908**, *330*, 377–445.

(5) Hulst, H. C.; van de Hulst, H. C. *Light scattering by small particles*; Courier Corporation, 1957.
